# MEK1/2 Inhibitors Unlock the Constrained Interferon Response in Macrophages Through IRF1 Signaling

**DOI:** 10.3389/fimmu.2019.02020

**Published:** 2019-08-27

**Authors:** Lei Yang, Jeak Ling Ding

**Affiliations:** Department of Biological Sciences, Faculty of Science, National University of Singapore, Singapore, Singapore

**Keywords:** macrophage, MAPK, MEK inhibitor, TLR7 agonist, IRF1, interferon, reprogramming

## Abstract

Macrophages are immune sentinels essential for pathogen recognition and immune defense. Nucleic acid-sensing toll-like receptors like TLR7 activate tailored proinflammatory and interferon responses in macrophages. Here we found that TLR7 activation constrained itself and other TLRs from inducing interferon response genes in macrophages through MAPK kinase 1/2 (MEK1/2)-dependent IRF1 inhibition. Downstream of the MEK1/2-ERK pathway, TLR7-activated macrophages induced interleukin-10 (IL-10), a signal transducer and activator of transcription 3 (STAT3) signaling axis, which constrained the expression of interferon response genes, immunomodulatory cytokines, and chemokines. Nevertheless, MEK1/2 inhibitors unlocked an IRF1-interferon signature response in an NF-κB-dependent manner. Deficiency in interferon regulatory factor 1 (*Irf1*) completely abrogated the interferon response and prevented the reprogramming of macrophages into an immunostimulatory phenotype. As a proof of concept, combination treatment with a TLR7 agonist and MEK1/2 inhibitor synergistically extended the survival of wild-type but not *Irf1*-deficient melanoma-bearing mice. In a retrospective study, higher expression of *Irf1* and interferon response genes correlated with more favorable prognosis in patients with cutaneous melanoma. Our findings demonstrated how MEK1/2 inhibitor unlocks IRF1-mediated interferon signature response in macrophages, and the therapeutic potentials of combination therapy with MEK1/2 inhibitor and TLR7 agonist.

## Introduction

Macrophages express a variety of specialized pathogen-recognition receptors like toll-like receptors (TLRs) which recognize pathogen-associated molecular patterns derived from invading pathogens or indirectly from infected host cells (1, 2). Recognition of double-stranded RNA (dsRNA) by TLR3 and lipopolysaccharide (LPS) by TLR4 in macrophages activate proinflammatory cytokines and interferon regulatory factor 3 (IRF3)-induced interferon (3). In contrast, recognition of CpG DNA by TLR9 and single-stranded RNA (ssRNA) by TLR7 activate mainly proinflammatory cytokines in macrophages. However, IRF7 induce type I interferons in plasmacytoid dendritic cells (pDCs) (3, 4). Secreted interferons and cytokines stimulate the janus kinase (JAK)-signal transducer and activator of transcription (STAT) pathway to transcribe secondary response genes that are essential for innate and adaptive immunity during anti-viral and anti-tumor responses (5, 6).

TLR7-based adjuvants are therapeutically effective in both anti-viral and anti-tumor immune responses (7). TLR7 agonist, imiquimod, has been approved for treatment of superficial basal cell carcinoma, genital and perianal warts (8), the effects of which are manifested by its potent interferon-inducing capabilities in pDCs but not in macrophages (9, 10). TLR7 is essential for the immunogenicity of viral vaccines (11), based on which the adjuvants greatly enhance humoral immune responses after immunization (12, 13). TLR7-based adjuvants also enhance anti-tumor effector T cell activity (14, 15) and subvert immunosuppression mediated by regulatory T (Treg) cells (16) and tumor-associated macrophages (17). Therefore, a better understanding of TLR7 signaling in antigen-presenting cells like macrophage is needed to elucidate viral RNA sensing and further improve TLR7-based therapies.

The mitogen-activated protein kinase (MAPK) kinase (MEK)1/2-extracellular signal-regulated kinase (ERK) MAPK pathway is activated by the kinase tumor progression locus 2 (TPL2) during both pro- and anti-inflammatory TLR responses in macrophages (18). After LPS stimulation in macrophages, TPL2 promotes the expression of cytokines and chemokines including tumor-necrosis factor α (*Tnf-*α), interleukin-1 (*Il-1*), *Il-6* (19), *Il-10* (20), and chemokine receptors (21). Anti-microbial nitric oxide synthase (NOS) is also TPL2-dependent after activation of multiple TLRs (22). After TLR4 or TLR9 activation, interferon-β (*Ifn-*β) expression is elevated in *Tpl2*^−/−^ macrophages, but not in pDCs (20, 23), which suggests that interferon responses may be constrained by TPL2 in some cell types. During TLR3 and TLR7 crosstalk, sustained ERK MAPK phosphorylation correlates with cytokine synergy and IRF1 suppression (24). After inhibition of MEK1/2 pathway, the synergistic production of cytokines IL-12, IL-6, TNF-α, and IL-10 are all reduced (24), whereas the effect on IRF1 suppression remains unknown. Given that inhibition of MEK1/2 pathway elevates IRF1-dependent interferon response in *RAS*-transformed fibroblasts (25) and cancer cells (26, 27), whether and how activated MEK1/2 pathway inhibits IRF1 to constrain the interferon response in cell types like keratinocyte (28) and TLR-activated macrophage (29) awaits further scrutiny. Because IRF1 is essential for both interferon production (30) and signaling (31), IRF1 may not be the sole interferon response gene tightly regulated by TLR7 signaling. A better understanding of the immunomodulatory roles of MEK1/2 pathway is warranted to harness versatile therapeutic potentials of interferons.

Here we examined whether TLR7 promotes inflammatory responses but constrains the transcription of interferon response genes like *Irf1* in macrophages. We found that interferon response in macrophages was inhibited by TLR7 activation, which depended on MEK1/2 activity. Concurrent TLR7 activation and MEK1/2 inhibition reprogrammed macrophages into an immunostimulatory phenotype through the NF-κB-IRF1 signaling axis. Combination treatment with TLR7 agonist and MEK1/2 inhibitor synergistically improved the survival of a murine melanoma model. Altogether, our findings offer mechanistic insights into how TLR activation prevents interferon responses in macrophages, and provide proof-of-concept evidence on how to augment interferon response to improve immune checkpoint blockade-based therapies or other anti-tumor immunotherapies.

## Results

### TLR7 Activation Constrains Itself and Other TLRs From Inducing Interferon Response Genes in Macrophages

**Figure 1:**
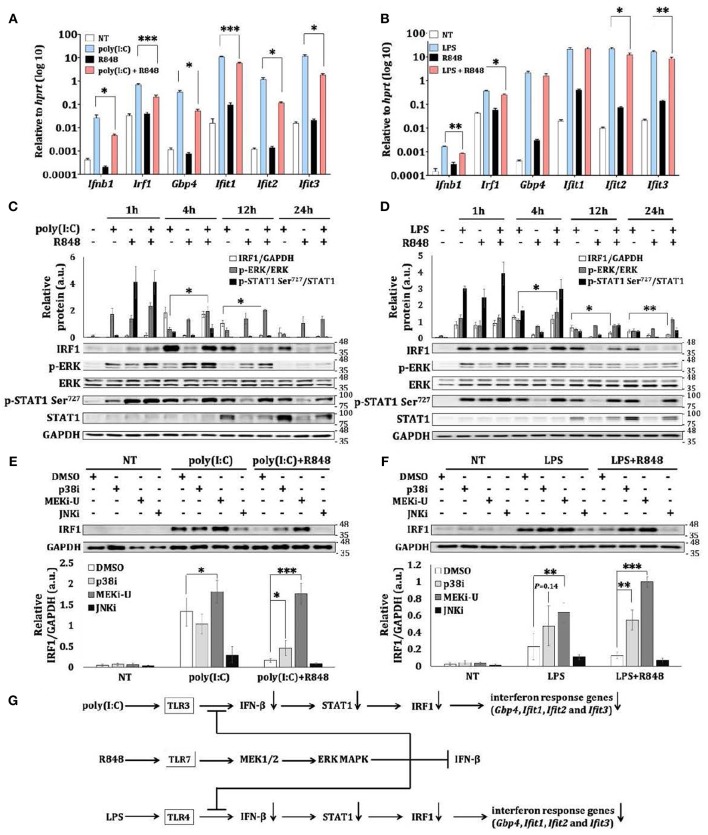
TLR7 stimulation constrains expression of interferon response genes during TLR crosstalk in macrophages. **(A,B)** qRT-PCR analysis of *Ifn-*β*, Irf1, Gbp4, Ifit1, Ifit2*, and *Ifit3* mRNA expression in BMDM stimulated as indicated for 12 h. Data are means ± SD from 4 experiments. **(C,D)** Immunoblot analysis and quantitative densitometry of IRF1, total and p-ERK, total and p-STAT1 in BMDM stimulated for indicated time intervals. Blots are representative of 3 or 4 experiments. Quantified data are means ± SD from all experiments. **(E,F)** Immunoblot analysis and quantitative densitometry of IRF1 in stimulated BMDMs stimulated with TLR3 agonist poly(I:C) and TLR7 agonist R848 **(E)**, or with TLR4 agonist LPS and R848 **(F)** for 12 h in the presence or absence of indicated MAPK inhibitors. Blots are representative of 4 or 5 experiments. Molecular weight (kDa) markers are indicated on the right side of the blots. Quantified data are means ± SD from all experiments. **(G)** Schematic illustration of TLR7-specific suppression on TLR3- and TLR4- induced interferon response and induction of intetferon response genes like *Irf1*. TLR7 also activates the MEK1/2-ERK MAPK pathway to limit itself from inducing interferon response. *P < 0.05, **P < 0.01, and ***P < 0.001 by unpaired Welch's *t*-test.

TLR7 activation of macrophages does not induce comparable amount of interferons as it does in pDCs (3, 4). We first utilized interferon-inducing TLR3 agonist poly(I:C) and TLR4 agonist LPS to study the crosstalk effects of TLR7 signaling in macrophages. During TLR3 and TLR7 crosstalk, interferon response gene, IRF1, is constrained (29). Consistently, we found that poly(I:C) but not TLR7 agonist R848 (24, 29) stimulated the expression of interferon response genes in bone marrow-derived macrophages (BMDMs) (Figure 1A). In contrast, co-treatment of macrophages with R848 and poly(I:C), or R848 and LPS significantly reduced the expression of interferon response genes including *Irf1* (Figures 1A,B). Besides IRF1, TLR7 activation also suppressed poly(I:C)- and LPS-activated total STAT1 (Figures 1C,D), which is indispensable for interferon signaling (5). Therefore, TLR7 may mount a general suppressive signaling to constrain the interferon response. This suppression was absent in macrophages deficient in TLR7 adaptor, *Myd88* (myeloid differentiation primary response 88) (Figures S1A,B), which suggests the direct involvement of a TLR7-specific mechanism.

MAPKs are known to mediate both the activation and suppression of TLR signaling (18). Consistently, we found an inverse relationship between TLR7-mediated suppression and phosphorylation of ERK MAPK (p-ERK) (Figures 1C,D). Target-specific inhibition of MEK1/2 pathway by MEK1/2 inhibitor, U0126 (MEKi-U) (26), but not inhibition of JNK MAPK (JNKi) (24, 29), resulted in the rescue of poly(I:C)- and LPS-activated IRF1 (Figures 1E,F). Although elevated IRF1 was also observed after inhibition of p38 MAPK (p38i), TLR7 does not activate p38 MAPK in a sustained manner (24), and therefore, does not correlate with TLR7-specific suppression. This suggests that the effect of p38 MAPK was mediated through a distinct mechanism as further demonstrated below. Because MEKi-U treatment did not alter mRNA stability (Figure S1C) and protein stability (Figure S1D) of IRF1 after TLR7 activation, the changes of *Irf1* mRNA expression may result in corresponding changes of IRF1 protein and IRF1-mediated functions as suggested before (32, 33). Collectively, our findings showed that a TLR7-specific signaling axis constrains TLR3- and TLR4-activated interferon responses (Figure 1G) in macrophages, presumably through the MEK1/2 pathway.

### MEK1/2 Inhibitor Synergizes With TLR7 Agonist to Unlock an Interferon Response Gene Signature

**Figure 2:**
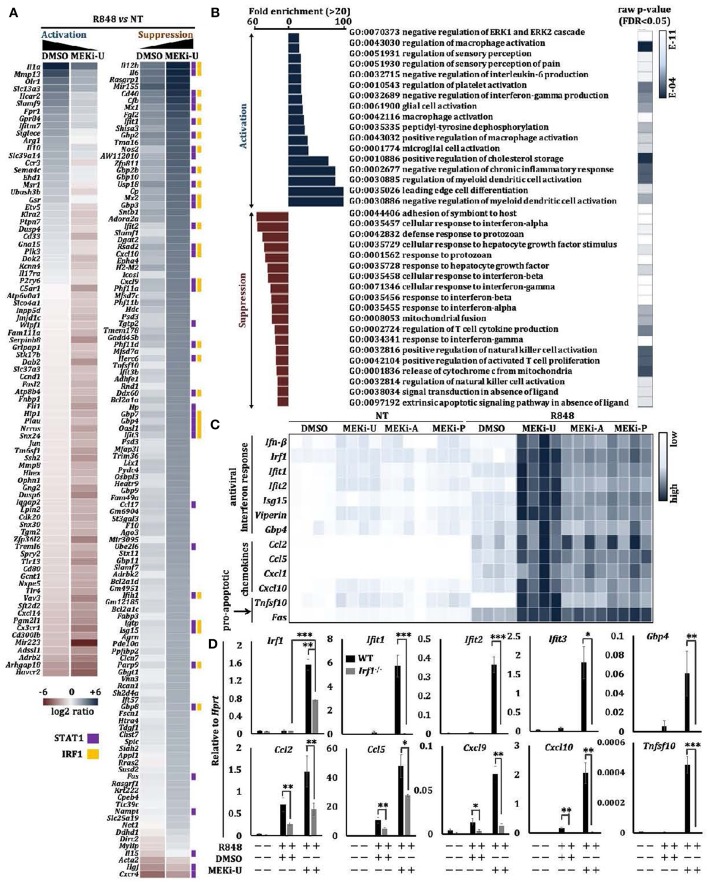
MEK1/2 inhibitor synergizes with TLR7 agonist to unlock an interferon signature response. **(A,B)** Microarray analysis of BMDMs treated with TLR7 agonist R848 in the presence or absence of MEKi-U for 6 h (see also Figure S2A). Heat map **(A)** and Gene set enrichment analysis **(B)** of differentially expressed genes are from 2 independent experiments. Differentially expressed genes interacting with STAT1 and IRF1 were labeled with purple and orange squares based on STRING PPI test. **(C)** Heat map showing qRT-PCR analysis of the mRNA expression of selected interferon response genes, chemokines, and pro-apoptotic genes. Data are from 4 independent experiments. **(D)** qRT-PCR analysis of interferon response genes, chemokines, and pro-apoptotic genes in WT and *Stat1*^−/−^ BMDMs treated with TLR7 agonist R848 in the presence or absence of MEKi-U for 6 h. Data are means ± SD from 4 independent experiments. *P < 0.05, **P < 0.01, and ***P < 0.001 by unpaired Welch's *t*-test.

To elucidate a general profile of TLR7-mediated suppression, we used whole transcriptome microarray analysis to identify genes differentially expressed in macrophages treated with R848 in the presence or absence of MEKi-U (Figure S2A). Compared to vehicle control, there were 32% more differentially expressed genes after MEK1/2 inhibition (Figure S2B), which were then shortlisted into Activation and Suppression categories (Figure 2A). Amongst genes that were suppressed by the MEK1/2 pathway, 33 and 24% interacted with STAT1 (Figure 2A, purple) or IRF1 (Figure 2A, orange), respectively. Using Gene Ontology (GO)-based gene set enrichment analysis, we found that the most highly enriched gene sets activated by MEK1/2 pathway were related to the inhibition of myeloid cell activation and response (Figure 2B, Activation). In contrast, the suppressed genes were highly enriched in gene sets exhibiting an “interferon signature response” (Figure 2B, Suppression).

To verify the above findings, we measured interferon response genes after TLR7 stimulation and MEK1/2 inhibition in macrophages, using three clinically tested MEK1/2 inhibitors: MEKi-U, MEKi-A (AZD6244, Selumetinib) (34, 35), and MEKi-P (PD0325901) (35). We found that MEK1/2 inhibition promoted the expression of interferon response genes (*Ifn-*β, *Irf1, Ifit1, Ifit2, Isg15, Viperin*, and *Gbp4*), chemokines (*Ccl2, Ccl5, Cxcl1*, and *Cxcl10*), and pro-apoptosis genes (*Tnfsf10* and *Fas*) (Figure 2C). In contrast, expression of the pro-inflammatory gene *Il-1*α was significantly reduced (Figure S2C) (20). Significantly elevated *Irf1* expression was also observed when TLR7 natural ligand, RNA40 (a U-rich single-stranded RNA derived from the HIV-1 long terminal repeat), but not RNA41 (derived from RNA40 by replacement of all uracil nucleotides with adenosine) was used to activate macrophages (Figure S2D). Notably, the p38 MAPK was unable to unlock the interferon signature response because its inhibitor (p38i) did not elevate or only mildly elevated mRNA expression of *Il-6* and interferon response genes (*Ifn-*β, *Irf1, Ifit1, Ifit2, Ifit3, Isg15, Viperin*, and *Gbp4*) (Figure S2E). These findings demonstrate how MEK1/2 inhibitor unlocks the interferon response in TLR7-stimulated macrophages.

### Enhanced Type I Interferon Signaling Unlocks the Interferon Signature Response

We then investigated whether and how interferons might mediate the unlocking of interferon signature response, and found that MEK1/2 inhibitor treatment did not alter *Ifn-*α expression in TLR7-activated macrophages (Figure S2F), which suggests the effects of MEK1/2 inhibition may act downstream of type I interferon. When we blocked the receptors for type I interferons (IFNAR) or type II interferon (IFNGR) during macrophage treatment with MEK1/2 inhibitor and TLR7 agonist, we found that STAT1 activation was only reduced by IFNAR neutralization (Figure S2G). These data suggested that the synergistic effects of MEK1/2 inhibition during TLR7 activation required type I interferon signaling. As compared to wild-type (WT) macrophages, R848-stimulated *Stat1*^−/−^ macrophages were defective in type I interferon signaling (4). After MEK1/2 inhibition, these macrophages expressed significantly lower or even abrogated levels of interferon response genes, including *Irf1* and chemokines (*Ccl2, Ccl5, Cxcl9*, and *Cxcl10*) (Figure 2D). Consistently, we did not detect any elevation of mRNA expression of other interferon response genes (*Ifit1, Ifit2, Ifit3*, and *Gbp4*) and pro-apoptosis gene *Tnfsf10* (Figure 2D). However, mRNA expression of *Fas* and *Cxcl1* remained unaltered (Figure S2H). These observations suggest the essential but selective role of the IFN-β-STAT1 pathway in the unlocked interferon signature response and the involvement of additional signaling axes.

### Unlocked IRF1 Mediates the Interferon Signature Response

We next profiled the mediator of the unlocked interferon signature response. In macrophages, interferons are known to activate an autocrine amplification loop directly through STAT1-STAT2-IRF9 complex and STAT1-STAT1 homodimers, or indirectly through transcription factors like IRF1 (31). IRF1 reciprocally induces type I interferons (30), suggesting that it may act both downstream and upstream of the interferon-STAT1 axis. We first tested its downstream functions using recombinant IFN-β and IFN-γ. Only IFN-β- but not IFN-γ- activated IRF1 was suppressed by TLR7 agonist (Figure S3A). Similarly, co-neutralization of IFNAR but not IFNGR inhibited the unlocked IRF1 (Figure S3B), indicating that IRF1 was activated at least in part by the IFN-β signaling. We then reconstituted STAT1 protein in *Stat1*^*inducible*^ macrophages (36) and observed a slight increase in *Irf1* expression (Figures S3C,D). However, this expression was not comparable to that observed after MEK1/2 inhibition (Figure 2C), which suggests that STAT1 probably plays only a partial role and may not be a major contributor in *Irf1* expression.

**Figure 3:**
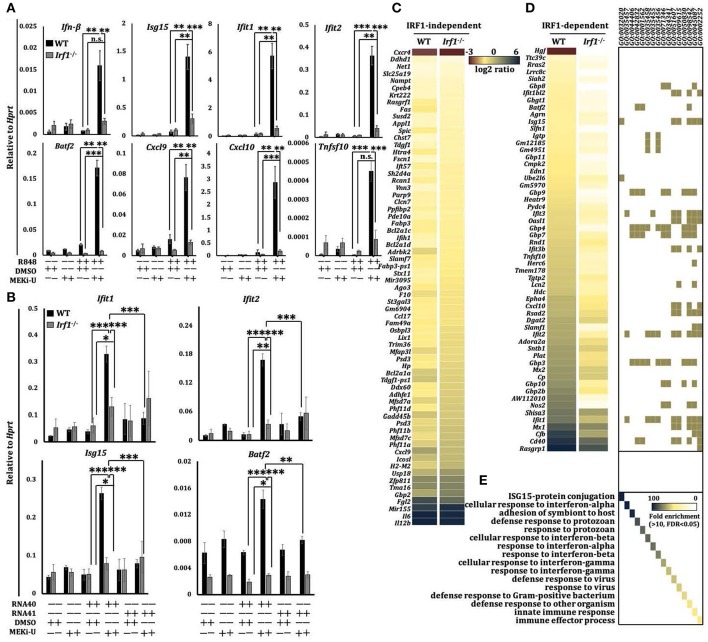
Unlocked interferon signature response is IRF1-dependent. **(A)** qRT-PCR analysis of mRNA expression of the indicated interferon response genes in WT and *Ifit1*^−/−^ BMDM stimulated with TLR7 agonist R848 for 6 h in the presence or absence of MEK1/2 inhibitor (MEKi-U). Data are means ± SD from 3 to 5 independent experiments. **(B)** qRT-PCR analysis of *Ifil1, Ifit2, Isgl5*, and *Batj2* mRNA expression in BMDM stimulated with RNA40 or RNA41 for 6 h in the presence or absence of MEKi-U. Data are means ± SD from 4 independent experiments. **(C–E)** Microarray analysis of mRNA expression in WT and *Ifit1*^−/−^ BMDM stimulated with TLR7 agonist R848 for 6 h in the presence or absence of MEKi-U. Heat map of IRF1-independent **(C)** or dependent **(D)** genes clustered using 1.5-fold difference cutoff. Gene set enrichment analysis of differentially expressed genes **(E)** are from all replicates. Quantified data are means ± SD from all experiments. *P < 0.05, **P < 0.01, and ***P < 0.001 by unpaired Welch's *t*-test. n.s., not significant.

Although TLR7 induced a transient expression of *Irf1* shortly after stimulation (Figure S3E), it did not persist. These data were consistent with the suppressed interferon response in TLR7-activated macrophages especially at later time points (Figure 2C). Indeed, compared to WT counterparts, the unlocked expression of all interferon response genes tested before and after MEKi-U treatment in R848- or RNA40-stimulated *Irf1*^−/−^ macrophages were significantly dampened (Figures 3A,B and Figure S3F). As expression of some but not all genes were still slightly elevated after MEK1/2 inhibition in *Irf1*^−/−^ macrophages (Figure 3A and Figure S3F), other molecules besides IRF1 may also play a role. We then used whole transcriptome analysis (Figure S3G) to compare differentially expressed genes between the WT and *Irf1*^−/−^ macrophages (Figure 3C). The gene set enrichment analysis showed that IRF1-dependent genes were highly enriched in interferon response-related gene sets (Figures 3D,E). These enriched gene set largely overlapped with those identified for the unlocked interferon signature response (Figure 2B), indicating that IRF1 unlocks the interferon signature response, at least in part, by mediating an autocrine IRF1-interferon-STAT1-IRF1 signaling axis.

### IRF1 Is Unlocked Through the Activation of NF-κB Pathway

**Figure 4:**
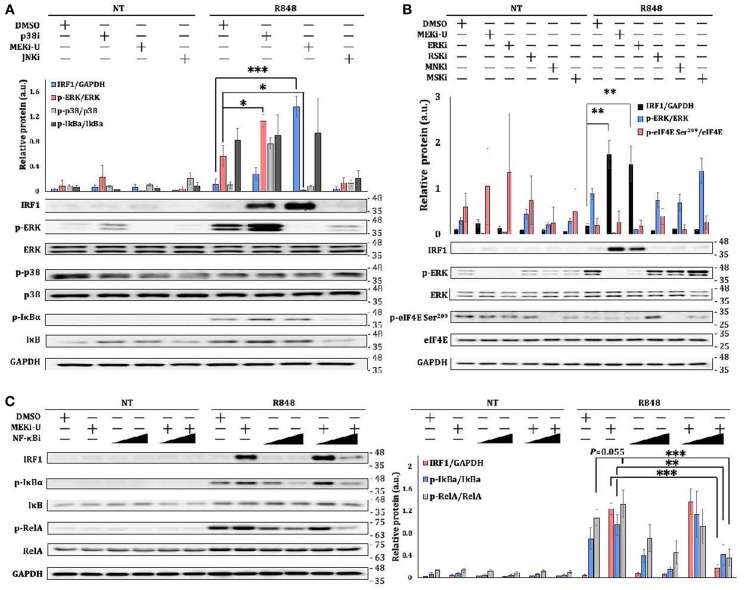
IRF1 is unlocked through the activation of NF-κB pathway. **(A–C)** Immunoblot analysis and quantitative densitometry of the indicated proteins in lysates from BMDM activated with R848 in the presence or absence of MEKi-U, p38 MAPK inhibitor p38i, or JNK MAPK inhibitor JNKi **(A)**, or MEKi-U, ERK inhibitor (ERKi), RSK inhibitor (RSKi), MNK inhibitor (MNKi), or MSK inhibitor (MSKi) **(B)**, or MEKi-U, and NF-κB inhibitor NF-κBi **(C)** for 12 h **(A,C)** or 8 h **(B)**. Blots are representative of 4 independent experiments. Molecular weight (kDa) markers are indicated on the right side of the blots. Quantified data are means ± SD from all experiments. *P < 0.05, **P < 0.01, and ***P < 0.001 by unpaired Welch's *t*-test. n.s., not significant.

That STAT1 only plays a partial role in promoting the expression of *Irf1* suggests that combination treatment may stimulate additional *Irf1*-inducing signaling axis. Because inhibition of NF-κB pathway restores inflammatory and interferon-based anti-tumor immunity (37, 38), we set out to investigate the involvement of NF-κB pathway. In line with the unlocked mRNA expression of *Irf1*, the IRF1 protein was also significantly elevated after MEK1/2 inhibition in TLR7-activated macrophages (Figure 4A). Although similar but much milder elevation of IRF1 was observed after treatment with p38i, a significant activation of ERK MAPK was identified (Figure 4A), further corroborating that p38i elevated IRF1 through a distinct mechanism. After treatment with multiple kinase inhibitors, only ERK inhibitor (ERKi, SCH 772984) (39) was found to promote IRF1 in a way similar to that of MEKi-U (Figure 4B). Other kinase inhibitors targeting ribosomal s6 kinase (RSK) (RSKi, BI-D1870) (40), MAPK interacting protein kinase (MNK) (MNKi, eFT508) (41, 42), or mitogen and stress activated protein kinase (MSK) (MSKi, SB-747651A) (43) did not increase or decrease the IRF1 protein (Figure 4B). These findings demonstrated that MEK1/2 pathway constrains the interferon signature response in TLR7-activated macrophages by RSK-, MNK-, and MSK-independent mechanism to contravene IRF1-inducing signaling such as the NF-κB pathway. Indeed, the activities of the NF-κB pathway, as measured by the phosphorylation ratios of IκBα and central effector RelA, were both increased after combination treatment (Figure 4C). Whereas, MEKi-U promoted IRF1 production when administered together with TLR7 agonist, the effect was abolished during treatment with a combination of MEKi-U and NF-κB inhibitor (NF-κBi) (Figure 4C). Therefore, the NF-κB pathway plays a significant role in unlocking the IRF1-interferon signature response.

### IL-10 Signaling Mediates the TLR7-Specific Suppression of IRF1-Dependent Interferon Signature Response

**Figure 5:**
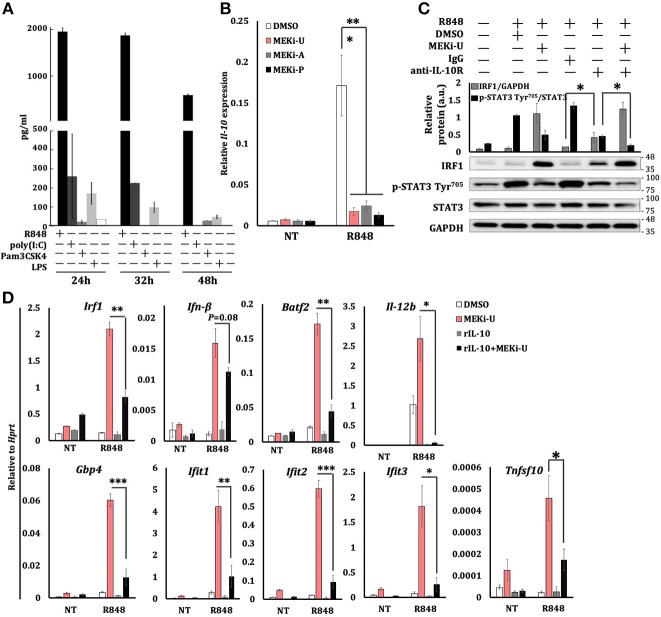
MEK1/2 pathway constrains IRF1-mediated interferon signature response through the IL-10 signaling. **(A)** ELISA analysis of IL-10 secretion from BMDM stimulated as indicated. Data are means ± SD pooled from 3 experiments. **(B)** qRT-PCR analysis of *Il-10* mRNA expression in BMDM stimulated with R848 for 6 h in the presence or absence of three MEK1/2 inhibitors. Data are means ± SD pooled from 4 experiments. **(C)** Immunoblot analysis and quantitative densitometry of IRF1, total, and p-STAT3 abundance in lysates of BMDM treated with R848 for 8 h in the presence or absence of MEKi-U, anti-IL-10R antibody, and IgG isotype control. Blots are representative of 4 independent experiments. Molecular weight (kDa) markers are indicated on the right side of the blots. Quantified data are means ± SD from all experiments. **(D)** qRT-PCR analysis of mRNA expression of the indicated genes in BMDM cells stimulated with R848 and recombinant IL-10 (20 ng/ml) for 6 h in the presence or absence of MEKi-U. Data are means ± SD pooled from 4 experiments. *P < 0.05, **P < 0.01, and ***P < 0.001 by one-way ANOVA **(B)** or unpaired Welch's *t*-test **(C,D)**.

We next examined the suppressive signaling which might be responsible for TLR7-specific suppression. We noted that IL-10-mediated inhibition is another hallmark of M2 macrophages (44). During TLR3-TLR7 crosstalk, we found that *Il-10* expression (Figure S4A) and STAT3 phosphorylation ratios (Figure S4B) were significantly elevated. Similar results were observed during TLR4-TLR7 crosstalk (Figure S4C). In TLR7-activated macrophages, we observed that *Il-10* expression peaked within an hour after stimulation, and it persisted inversely with the kinetics of TLR7-specific suppression (Figure S4D). Remarkably, R848 stimulation produced the largest amount of IL-10 protein compared to other TLR agonists, including poly(I:C), Pam3CSK4 (TLR1/2 agonist), and LPS (Figure 5A). Furthermore, after treatment with all three MEK1/2 inhibitors, the TLR7 activation failed to induce *Il-10* (Figure 5B). However, treatment with p38i induced *Il-10* (Figure S4E). This functional discrepancy between MEK1/2 pathway and p38 MAPK pathway plausibly explained their distinct effects on the interferon signature response. Notably, *Stat1*^−/−^ macrophages displayed significantly reduced *Il-10* expression, regardless of MEK1/2 inhibition (Figure S4F), indicating an IL-10-mediated negative feedback loop downstream of the interferon-STAT1 signaling, as suggested before (45).

During combination treatment, neutralization of the IL-10 receptor (IL-10R) decreased STAT3 phosphorylation ratios, which correlated with increased IRF1 abundance (Figure 5C). Similar results were observed during TLR3-TLR7 crosstalk (Figure S4G). However, MEK1/2 inhibitor treatment did not alter TLR7-induced *Il-10* expression when added even an hour after R848 stimulation (Figure S4H), which clearly explains the expression kinetics of *Irf1* (Figure S3E). Notably, the addition of recombinant IL-10 significantly inhibited the expression of interferon response genes unlocked by MEK1/2 inhibitor (Figure 5D). These data suggest that TLR7-MEK1/2-ERK MAPK-IL-10-STAT3 axis mediates the suppression of NF-κB-IRF1-induced interferon signature response in macrophages.

### Combination Treatment Reprograms Macrophages Toward a Predominantly M1-Like Phenotype Through an IRF1-Interferon-STAT1 Pathway

**Figure 6:**
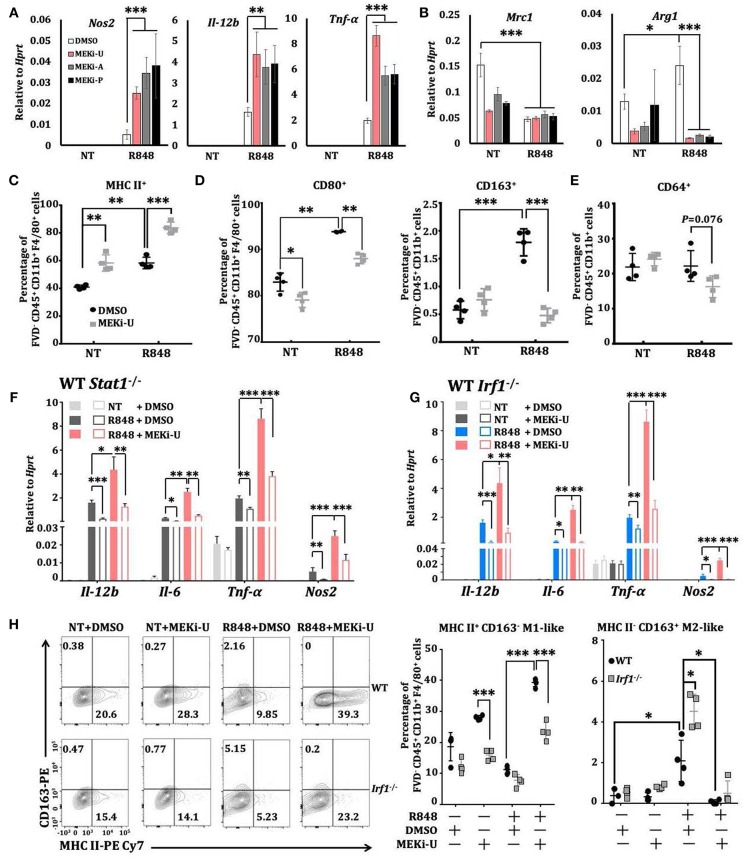
Macrophages are reprogrammed toward a predominantly M1-like phenotype through IRF1. **(A,B)** qRT-PCR analysis of *Nos2, Il-12b*, and *Tnf*α **(A)** or *Mrc1* and *Arg1* **(B)** mRNA expression in BMDMs activated with TLR7 agonist R848 for 6 h in the presence or absence of different MEK1/2 inhibitors. Data are means ± SD pooled from 4 experiments. **(C–E)** Flow cytometry analysis of MHC II **(C)**, CD80 and CD163 **(D)**, and CD64 **(E)** surface expression on BMDMs stimulated with R848 for 24 h in the presence or absence of MEKi-U. Data with means ± SD are from 4 independent experiments. **(F,G)** qRT-PCR analysis of the indicated mRNAs expressed in WT and *Stat1*^−/−^ **(F)** or *Irf1*^−/−^ **(G)** BMDMs activated with TLR7 agonist R848 for 6 h in the presence or absence of different MEK1/2 inhibitors. Data are means± SD pooled from 4 experiments. **(H)** Flow cytometry analysis of MHC II^+^ CD163^−^ M1 and MHC II^−^ CD163^+^ M2 phenotypes in WT and *Irf1*^−/−^mice BMDM stimulated with R848 for 24 h in the presence or absence of MEKi-U. Contour plots are representative of 4 independent experiments. Quantified% positive data with means ± SD are from all experiments. *P < 0.05, **P < 0.01, and ***P < 0.001 by one-way ANOVA **(A,B)** with Bonferroni's correction **(F,G)** or unpaired Welch's *t*-test **(C–H)**.

Macrophages are highly versatile and undergo rapid phenotype reprogramming in response to dynamic environmental cues (44). While classically activated M1 macrophages are immunostimulatory, alternatively activated M2 macrophages are immunosuppressive (44). Notably, significant increases of M1 macrophage phenotype markers, *Il-12b* and *Il-6*, were observed after treatment with MEK1/2 inhibitor and TLR7 agonist (Figure S2E). We hypothesized that combination treatment may alter the M2-like phenotype of *in vitro* M-CSF generated BMDMs through IRF1-mediated interferon signature response or *vice versa*. To test this idea, we examined the phenotype of macrophages activated by R848 or RNA40 in the presence or absence of MEKi-U and found a mixed phenotype. There were increased expressions of both M1 phenotype markers (Figure 6A and Figure S5) and M2 phenotype markers, *Arg1* and *Mrc1* (also known as CD206) (Figure 6B). Similar results were observed for M1 marker MHC II (Figure 6C) and M2 markers CD80 (high-affinity ligand for immune checkpoint CD152/CTLA-4), CD163, and CD64 (Figures 6D,E). In contrast, MEK1/2 inhibitor altered only two markers, including MHC II and CD80, but not others (Figures 6A–E). During combination treatment, macrophages predominantly exhibited M1-like phenotype with elevated M1 markers (Figures 6A,C) and decreased M2 markers (Figures 6B,D,E). Deficiencies of both *Stat1* (Figure 6F) and *Irf1* (Figure 6G and Figure S5) reduced the elevated expression of all four M1 markers. Consistently, *Irf1* deficiency constrained M1-like macrophages (MHC II^+^ CD163^−^) but promoted M2-like macrophages (MHC II^−^ CD163^+^) (Figure 6H), suggesting that IRF1 has a role in macrophage reprogramming.

### Combination Treatment With MEK1/2 Inhibitor and TLR7 Agonist Improves Animal Survival in a Murine Melanoma Model

**Figure 7:**
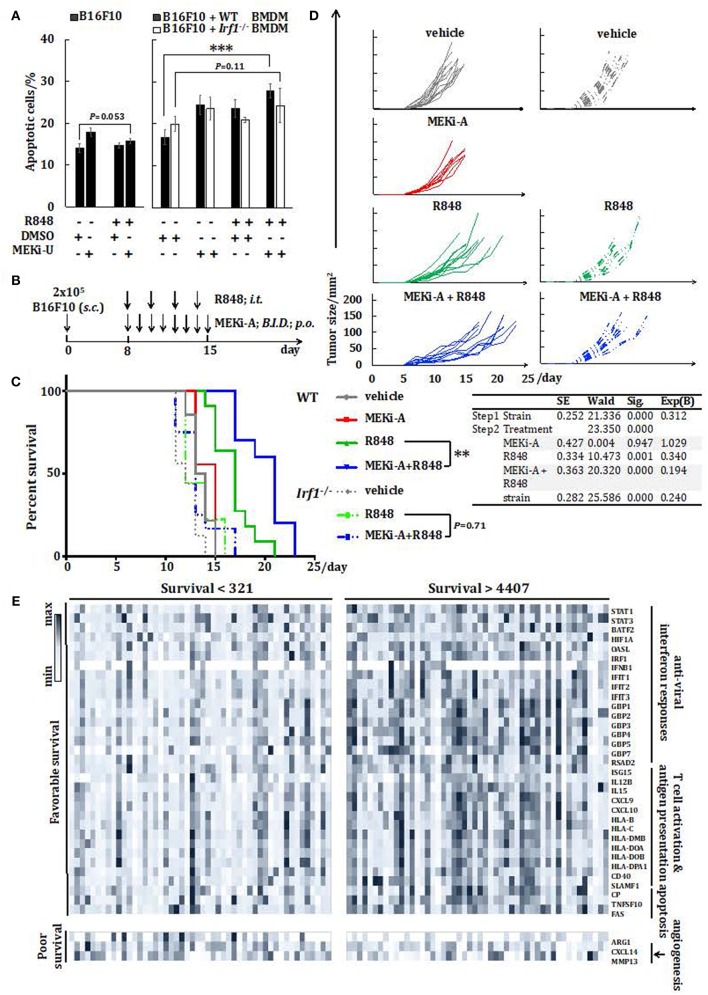
Combination therapy with MEK1/2 inhibitor and TLR7 agonist reduces melanoma progression *in vivo*. **(A)** Flow cytometry analysis of B16F10 apoptosis cultured alone or co-cultured with WT or *Irf1*^−/−^ BMDM. Cells were stimulated with R848 in the presence or absence of MEKi-U for 48 h. Data are means ± SD from 4 independent experiments. **(B–D)** WT or *Irf1*^−/−^ mice bearing subcutaneous (s.c.) B16F10 melanoma tumors were treated with MEKi-A or intratumoral (i.t.) R848 or both as indicated in **(B)**. **(C,D)** Survival analysis **(C)** and tumor growth **(D)** of WT or *Irf1*^−/−^ melanoma-bearing mice treated with MEKi-A and/or R848. Data from at least 9 mice/group are pooled from 2 to 3 independent experiments. **(E)** Heat map showing mRNA expression of 36 genes identified with significant associations between their expression levels and patient survival. Data presented are from individuals with overall survival shorter than 321 days (*n* = 50) or longer than 4,407 days (*n* = 50). *P < 0.05, **P < 0.01, ***P < 0.001 by unpaired Welch's t-test **(A)**, Cox regression analysis **(C)**, or log-rank test **(C,E)**.

Since macrophages can be tumoricidal through phagocytic and immunostimulatory roles in the tumor microenvironment (44), we next explored the therapeutic potentials of the unlocked interferon signature responses *in vitro*. Combination treatment with MEK1/2 inhibitor and TLR7 agonist did not promote or reduce apoptosis of B16F10 melanoma cells cultured alone (Figure 7A). However, during co-culture with activated WT but not *Irf1*^−/−^ BMDM, apoptosis of B16F10 was significantly elevated (Figure 7A). This was consistent with the above *in vitro* studies showing that combination of TLR7 agonist and MEK1/2 inhibitor reprogrammed macrophages into anti-cancer M1-like phenotype.

Based on our *in vitro* studies, we postulated that MEK1/2 inhibitor may boost TLR7-mediated anti-melanoma interferon responses *in vivo*. As a proof of concept, we probed the therapeutic potentials of the combination of MEK1/2 inhibitor and TLR7 agonist in a murine model of subcutaneous melanoma. The melanoma-bearing WT mice were treated with either TLR7 agonist or MEK1/2 inhibitor, or a combination of TLR7 agonist and MEK1/2 inhibitor (Figure 7B). Compared to vehicle controls, single treatment with MEK1/2 inhibitor did not alter animal survival, possibly due to its selective effects on *RAS*/*RAF*-mutated cancers (46). In contrast, TLR7 agonist alone delayed mortality and slowed melanoma growth (Figures 7C,D). However, the combination treatment significantly improved the animal survival and retarded melanoma progression when compared to TLR7 agonist single treatment (Figures 7C,D). Thus, TLR7 agonists may synergize with MEK1/2 inhibitor to augment the anti-tumor treatment efficacy.

To directly test the involvement of IRF1, we treated both the WT and *Irf1*^−/−^ mice with TLR7 agonist and the combination treatment. In line with the tumorigenic susceptibility of *Irf1*^−/−^ mice (47), significant reduction in survival was observed in untreated *Irf1*^−/−^ mice than in WT controls (Figures 7C,D). Both single treatment with TLR7 agonist and the combination treatment largely extended the survival of *Irf1*^−/−^ mice, albeit with significantly reduced effects when compared to WT mice (Figures 7C,D). Remarkably, we did not observe any difference in survival of *Irf1*^−/−^ mice between single treatment and combination treatment (Figure 7C), suggesting that IRF1 is needed for the synergistic interferon signature response.

### The Interferon Signature Response Indicates Favorable Prognosis in Melanoma Patients

**Figure 8:**
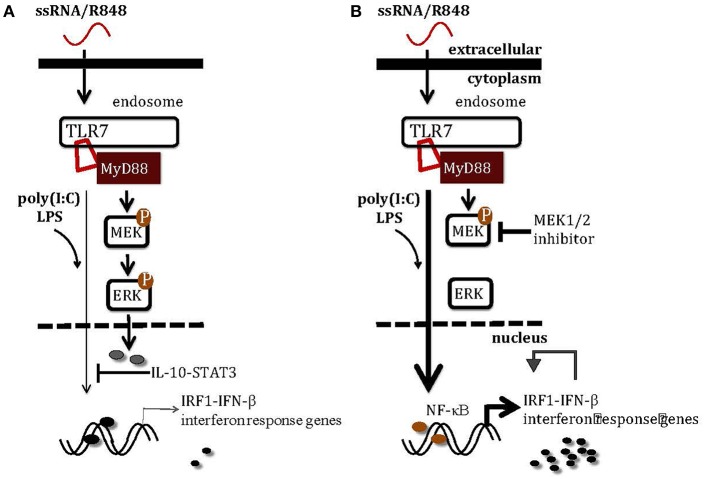
A schematic illustration of how MEK1/2 pathway modulates the interferon responses in macrophages. **(A)** TLR3 activation by poly(I:C) and TLR4 activation by LPS induce scarce levels of *Irf1* and interferon response genes, at least in part due to the inhibitory effects of MEK1/2-ERK-IL-10-STAT3 signaling. **(B)** Inhibition of MEK1/2 pathway using MEK1/2 inhibitor blocks activation of ERK-mediated inhibitory signaling, unlocking the IRF1-IFN-β-interferon signature response through the NF-κB pathway. The combination therapy with TLR7 agonist and MEK1/2 inhibitor may greatly enhance the anti-tumor monotherapies with either TLR7 agonist or MEK1/2 inhibitor by boosting the interferon signature response.

To gain better insights into the translational potentials of the combination treatment, we analyzed the association between interferon response genes and 5-year survival of cutaneous melanoma patients. Thirty-six genes that are well-known cancer-associated genes or have been verified in this study, were found to be associated with either significantly favorable or poorer 5-year survival rates. In particular, eighteen interferon response genes, twelve T cell activation and antigen presentation-related genes, and three pro-apoptosis genes showed positive associations with favorable survival rates (Table S1 and Figure 7E). On the other hand, the M2 macrophage phenotype gene, *Arg1*, and two pro-angiogenesis/tumor genes (*Cxcl14* and *Mmp13*) were identified as poor prognosis markers (Figure 7E). We next examined individual expression profiles of the above-mentioned genes in representative patient samples. Patients with the longest overall survival (>4407 days vs. <321 days) harbored higher expression of genes involved in the interferon signature response, T cell activation and antigen presentation, apoptosis, but not angiogenesis (Figure 7E). These findings demonstrated potentials of interferon signature response-based prognosis in melanoma patients and importantly, the implications of our combination treatment strategy (Figure 8).

## Discussion

Our findings demonstrated that interferon responses are restricted in macrophages after TLR7 activation. As aberrant TLR7 signaling and type I interferons can cause autoimmune disorders (47) and lethal inflammation (48), TLR7-activated macrophages may be genetically programmed to prevent excessive production of type I interferons but not the proinflammatory responses. Indeed, macrophage-generated cytokines are essential for the recruitment and activation of additional immune cells during inflammation and immune defense (5, 6). In contrast, interferon response genes became detectable in TLR7-activated macrophages only after MEK1/2 inhibition, consistent with TPL2-mediated interferon suppression in TLR4- and TLR9-activated macrophages (20). Intriguingly, interferon responses in pDCs are not affected by Tpl2 deficiency (18, 20) and are even enhanced by MEK1/2 inhibition (49), suggesting that the TLR7-interferon signaling may be differentially activated in macrophages and specialized interferon-producing pDCs.

Recently the FDA-approved inhibitors targeting the RAS-RAF-MEK1/2-ERK pathway have revolutionized treatment schemes for advanced-stage melanoma (35, 50). However, their antineoplastic effects are compromised by dose-limiting side effects (35, 50) and cancer-cell autonomous resistance (51). Alternative strategies are combination treatments incorporating both novel MEK1/2 inhibitors and immune cell-targeting agents (50, 51), of which the therapeutic potentials were demonstrated in our studies. In particular, inhibition of MEK1/2 pathway reprogrammed macrophages into immunostimulatory M1-like phenotype and increased macrophage apoptosis, which possibly reduces macrophage-mediated tumor-promoting effects. Mechanistically, the MEK1/2-ERK MAPK pathway either amplifies or dampens upstream signals by phosphorylating hundreds of substrates in a context-dependent manner (18). However, our studies found that the MEK1/2-ERK MAPK pathway did not activate its downstream kinases such as RSK, MSK, and MNK, but inhibited the NF-κB pathway to constrain IRF1-mediated interferon responses. A recent study has identified how herpes simplex virus type 1 induces microRNA-373 to suppress IRF1-mediated type I interferon (52). It would therefore be interesting to examine the specific substrate(s) involved in MEK1/2-ERK-mediated suppressive signaling, especially the potential involvement of novel regulatory microRNAs. We also noted that previous studies have shown the selective anti-tumor effects in *RAF*-mutated melanomas (46), which explains our observations that MEK1/2 inhibitor did not alter the apoptosis of B16F10 melanoma cells *in vitro* and animal survival *in vivo*. Therefore, the synergistic effects observed after combination treatment may be conceivably improved in *RAS*/*RAF*-mutated cancers.

Although treatment with TLR7 agonist alone significantly improved animal survival, its effects were greatly enhanced when combined with MEK1/2 inhibitor. It is possible that the therapeutic efficacy of TLR7 agonist is limited by its incompetency to overcome macrophage-mediated immunosuppressive roles coordinated by molecules like IL-10 (44). Indeed, TLR7 stimulation led to mixed phenotypes in macrophages that were reprogrammed to predominantly M1-like macrophages after MEK1/2 inhibition, despite the M2-like nature of *in vitro* M-CSF-generated primary macrophages. The synergistic roles of MEK1/2 inhibitor, when combined with TLR7 agonist, may be explained by: (i) subversion of the immunosuppressive roles of IL-10 signaling, (ii) regulatory roles of IRF1 in cellular metabolism, and (iii) IRF1-mediated gene expression of macrophage M1 markers such as TNF-α, a potent anti-cancer cytokine (53). To harness the therapeutic potentials of macrophages, at least four strategies have been proposed, including the elimination of macrophage at tumor sites, the prevention of macrophage recruitment from blood stream, the blockade of “don't eat me” signal and the reprogramming of macrophage phenotypes (44). Instead of overlooking the immunostimulatory potentials of macrophages, we showed here, how the immunosuppressive effects of macrophages can be subverted while retaining direct melanoma killing effects. As an alternative to the recombinant interferon therapy (54, 55) and pDC-based therapies (10, 56), our proof-of-concept findings propose an alternative strategy that may be used to target macrophages and mount the anti-tumor interferon responses.

Our studies suggested that MEK1/2 pathway constrained interferon responses in macrophages after TLR7 activation. However, whether MEK1/2 pathway ameliorates TLR7-related inflammation or autoimmune disorders by constraining interferon responses remains to be established. While our murine melanoma model demonstrated the efficacy of the combination treatment, whether and how this strategy generates even better preclinical outcomes in *RAS*/*RAF*-mutated cancers requires further investigation. It will also be interesting to elucidate how effector cell-targeting therapies like immune checkpoint blockades may improve our combination strategy. Nevertheless, the efficacies of our combination strategy are at least 4-fold: (I) reprogramming of immunosuppressive macrophages, (II) exploitation of the pleiotropic effects of interferons, cytokines, and pro-apoptotic molecules from reprogrammed macrophages, (III) maintenance of TLR7-based immunomodulatory effects in pDCs, and (IV) maintenance of MEK1/2 inhibitor-based antineoplastic roles in *RAS*/*RAF*-mutated cancers.

## Materials and Methods

### Animals

Age- and sex-matched mice were used in all experiments. WT C57BL/6JInv were purchased from InVivos Pte Ltd. B6.129S2-*Irf1*^*tm*1*Mak*^/J (*Irf1*^−/−^) and B6.129S(Cg)-*Stat1*^*tm*1*Dlv*^/J (*Stat1*^−/−^) were from Jackson Laboratories. Femurs and tibias of B6.129P2(SJL)-*Myd88*^*tm*1.1*Defr*^/J (*Myd88*^−/−^) were kindly provided by Dr. Norman Pavelka (Singapore Immunology Network, Singapore). *Stat1*^inducible^ mice were from Professor Mathias Muller (Inst. Of Animal Breeding and Genetics, University of Veterinary Medicine, Vienna). All experiments were carried out in accordance with institutional guidelines prescribed by the Institutional Animal Care and Use Committee, National University of Singapore (protocol number R13-5667 and R17-1205).

### Cell Culture

BMDMs were derived from the bone marrow cells using mouse recombinant M-CSF as described before (36). J774.1 macrophage cell line (ATCC) and B16F10 melanoma cells (ATCC^®^ CRL-6475™) were cultured in Dulbecco's modified Eagle's medium (Gibco, Thermo Fisher Scientific) supplemented with 10% (v/v) fetal bovine serum (HyClone, Thermo Fisher Scientific) and Pen-Strep (100 U/ml for penicillin and 100 μg/ml for streptomycin; Gibco, Thermo Fisher Scientific). BMDM and J774.1 cells were plated at 1 × 10^6^ and 0.4 × 10^6^ cells/ml, respectively. All cells were cultured at 37°C with 5% CO_2_.

### B16F10 and BMDM Co-culture

B16F10 cells were cultured at a density of 0.2 × 10^6^ cells/ml alone or co-cultured with BMDM cells derived from WT or *Irf1*^−/−^ mice at 1:1 B16F10/BMDM ratio for 2 days. Cells were then digested with TrypLE™ Express Enzyme (1×, Gibco, Thermo Fisher Scientific) for 3–5 min at 37°C before cell death and apoptosis analyses using flow cytometry. For co-culture transwell assay, B16F10 melanoma cells and BMDM were plated into culture plates and inserts with pore size of 0.4 μm, respectively. Only B16F10 melanoma cells were harvested for downstream analysis.

### Reagents and Antibodies

TLR agonists were used at 10 μg/ml for poly(I:C) (LMW; Invivogen), 25 ng/ml (*in vitro*) and 3 mg/kg (*in vivo*) for R848 (Invivogen), 5 μg/ml for ssRNA40/LyoVec™ and ssRNA41/LyoVec™ (Invivogen), 10 ng/ml for Pam3CSK4 (Calbiochem, EMD Biochemicals), and 100 ng/ml for LPS (Sigma-Aldrich). Recombinant mouse IL-10 (cat. no. 575802), IFN-β1 (cat. no. 581302) and IFN-γ (cat. no. 575302) proteins were purchased from Biolegend. MEKi-U (cat. no. #9903; Cell Signaling Technology), MEKi-A (Selleck, USA) and MEKi-P (Selleck, USA) were used at 10, 0.5, and 0.5 μM, respectively. mRNA synthesis and protein translation were inhibited by 5 μg/ml actinomycin D (Sigma-Aldrich) and 80 μg/ml cycloheximide (Sigma-Aldrich), respectively. p38i (cat. no. #5633) and JNKi (cat. no. #8177) used at 10 μM, were from Cell Signaling Technology. NF-κBi (sc-202490) was from Santa Cruz Biotechnology. ERKi (cat. no. #19166-1), MNKi (cat. no. 21957-1), and RSKi (cat. no. #501437-28-1) were from Cayman Chemical and used at 1, 1, and 5 μM, respectively. MSKi (cat. no. Axon-1897, Axon Medchem) was used at 5 μM. Antibodies (Table S2) were used at 1:1000 dilution unless otherwise specified (10 μg/ml for neutralization assays). Isotype controls, neutralizing antibodies, inhibitors (except for actinomycin D) were added 1 h before stimulations. For STAT1 reconstitution, BMDM from *Stat1*^inducible^ mice were incubated with doxycycline (cat. no. 17086-28-1; Sigma-Aldrich) as described before (36).

### Murine Melanoma Model

An inoculum of 0.2 million mycoplasma-free B16F10 (ATCC® CRL-6475™) cells were injected subcutaneously into the left flank of C57BL/6 mice. Seven days later, mice with palpable tumors were randomly divided into experimental groups. R848 was administered intratumorally at 3 mg/kg every 2 days. MEKi-A was formulated in sterile vehicle with 0.5% (hydroxypropyl)methyl cellulose (Sigma-Aldrich) and 0.1% polysorbate 80 (v/v) (Sigma-Aldrich) (26). Fifty mg/kg MEKi-A was administered twice daily by oral gavage. Control group was treated with vehicle twice daily and sterile phosphate-buffered saline at 3 ml/kg every 2 days. Tumor size was monitored as an area (longest dimension × perpendicular dimension) using caliper, thrice per week until experimental endpoints (tumor size > 100 cm^2^) were reached (57).

### Interferon Signature Response in the Cancer Genome Atlas (TCGA) Data

Associations between expression levels of 53 immune response genes and 5-year survival patient survival were analyzed. Clinical data for 462 TCGA skin cutaneous melanoma (SKCM) patients (58) were downloaded using UCSC Xena (http://xena.ucsc.edu/), including the overall survival in days and gene expression data from Illumia HTseq 2000 RNAseq platform (Table S1). The median follow-up from diagnosis recorded was 1,124 days with a range of 6–11,252 days. For each gene, we used a similar method as reported before (37) to score the gene expression above or below the median expression, and examined their associations with truncated 5-year survival (1,825 days) using log-rank tests. Genes with expression profiles associated with favorable survival (*P* < 0.05) were considered as positive prognosis markers.

### Flow Cytometry

Single-cell suspensions were stained with fixable viability dye eFluor™ 506 (ebioscience, Thermo Fisher Scientific), blocked with 2.5 μg/ml anti-CD16/32 (clone 93; ebioscience, Thermo Fisher Scientific), and then stained for cell surface markers. Cells were fixed and permeabilized using Foxp3/Transcription Factor Staining Buffer Set (ebioscience, Thermo Fisher Scientific) before intracellular staining. Antibodies are listed in Table S2. For cell death and proliferation assays, cells were stained with fixable viability dye eFluor™ 450 (ebioscience, Thermo Fisher Scientific) followed by intracellular staining of Ki67-PerCP-eFluor™ 710. For cell death and apoptosis assays, co-cultured cells were first stained with CD45- eFluor™ 450 to exclude BMDM followed by 7-AAD and Annexin-V-FITC in 1 × Annexin V binding buffer (ebioscience, Thermo Fisher Scientific) following the manufacturer's instructions. B16F10 cells alone or co-cultured in transwell assays were directly stained with 7-AAD and Annexin V-FITC before analysis. All samples were filtered through 60 μm cell strainers before analysis using BD LSRFortessa™ (BD Biosciences) and FlowJo software (FlowJo, LLC).

### Quantitative RT-PCR

Total RNA was purified using TRIzol™ reagent (Invitrogen, Thermo Fisher Scientific) and reverse transcribed using SuperScript III First-Strand Synthesis System (Thermo Fisher Scientific) according to the manufacturer's instructions. cDNA templates were then analyzed using GoTaq® qPCR Master Mix (Promega Corporation) on a LightCycler™ 480 Instrument II (Riche Life Science). Gene expression was normalized to housekeeping gene, hypoxanthine phosphoribosyltransferase 1 (*Hprt*). The primers are listed in Table S3.

### Western Blotting

Cell lysate was prepared using radioimmunoprecipitation assay buffer (1x RIPA buffer; 50 mM Tris pH7.5, 0.5% deoxycholate, 150 mM NaCl, 1% NP-40, 0.1% SDS, 1 mM EDTA) supplemented with protease inhibitor cocktail (Sigma-Aldrich). Cell lysate was then boiled for 10 min at 100°C in denaturing Laemmli buffer and analyzed using SDS-PAGE, followed by blocking with 5% skim milk (Sigma-Aldrich) and incubating with indicated primary antibodies. Blots were incubated with horseradish peroxidase–conjugated rabbit or mouse secondary antibodies (Sigma-Aldrich) and visualized using WesternBright ECL (Advansta) in an ImageQuant LAS 4000 mini system (GE Healthcare). Blots were re-probed for GAPDH loading controls after stripping using Restore™ Western Blot Stripping Buffer (Thermo Fisher Scientific). For densitometry analysis, ImageJ (NIH) was used following user guidebook.

### ELISA

IL-10 secretion was determined using the mouse IL-10 ELISA set (BD Biosciences) according to the manufacturer's instructions.

### RNA Purification for GeneChip®

RNA isolation was performed using TRIzol™ reagent (Invitrogen, Thermo Fisher Scientific) and RNeasy® Plus Mini Kit (Qiagen) according to the manufacturer's instructions. Genomic DNA was removed using Ambion™ DNase I (RNase-free) (Thermo Fisher Scientific) before elution. Quantities and qualities of purified RNA were assessed using NanoDrop Spectrophotometer (Thermo Fisher Scientific) and Agilent Bioanalyzer (Agilent Technology). Samples with no significant contamination and with RNA integrity numbers over 8.0 were included for downstream analysis.

### Whole Transcriptome Analysis

Double-stranded cDNA was generated, fragmented, and end-labeled using the GeneChip® WT PLUS Reagent Kit (Thermo Fisher Scientific). Labeled cDNA was then processed and hybridized to GeneChip® Mouse Transcriptome Array 1.0 (Affymetrix, Thermo Fisher Scientific) using AFX Fluidics 450 Station (Thermo Fisher Scientific). GeneChips were scanned on a GC3000 G7 Scanner (Thermo Fisher Scientific). Raw microarray files were then extracted, annotated and normalized using RMA algorithm of Partek Genomic Suite (Partek Incorporated). Probe set information was then log2 transformed and consolidated at gene levels. Analysis of variance (ANOVA) was performed with contrasts set against corresponding non-treated controls. Genes differentially expressed by no <1.5-folds with adjusted *P*-values < 0.05 unless otherwise specified were shortlisted for downstream analysis. For analysis of IRF1-dependent and -independent genes, differentially expressed genes identified in *Irf1*^−/−^ macrophages with 1.5-fold higher or lower expression than their WT counterparts were classified as IRF1-dependent or -independent, respectively.

### Gene Set Enrichment Analysis

Shortlisted genes were imported for PANTHER overrepresentation test using the default Fisher's Exact Test with false-discovery rate (FDR) correction for multiple tests (59, 60). Gene lists with FDR < 0.05, raw *P*-value < 10^−4^ and fold enrichment > 10, unless otherwise specified, were considered to be significantly enriched.

### STRING PPI Test

Differentially expressed genes were analyzed using STRING database (version 10.5) with default settings and minimum required interaction score (0.400) (61). The active interaction sources were customized to include only known interactions from curated databases and experimental data.

### Statistics

Data are presented as means ± SD. Statistical analysis was performed using Prism 6.0 software (Graph Pad Software), Microsoft Excel 2017 or IBM SPSS. Unpaired Welch's *t*-test, Cox regression analysis, log-rank test, one-way ANOVA, or one-way ANOVA with Bonferroni's correction for multiple comparisons were used to determine the statistical significances. *P*-value < 0.05 were considered significant. Researchers were not blinded to the experimental groups and all samples were included for analysis.

## Data Availability

The microarray datasets generated for this study can be found in the Gene Expression Omnibus (GEO) with GEO accession number GSE123512. Other raw data supporting the conclusion of this manuscript will be made available by the author upon request.

## Author Contributions

LY designed and performed all experiments. JD conceived and supported all studies. LY and JD analyzed the data and wrote the manuscript.

### Conflict of Interest Statement

The authors declare that the research was conducted in the absence of any commercial or financial relationships that could be construed as a potential conflict of interest.
