# Supplementary material for: MEK1/2 Inhibitors Unlock the Constrained Interferon Response in Macrophages Through IRF1 Signaling
Source: Front Immunol. 2019 Aug 27;10:2020. doi: 10.3389/fimmu.2019.02020 (PMC6718554; doi:10.3389/fimmu.2019.02020)
Supplement: Supplementary file 1 [file Data_Sheet_1.doc]

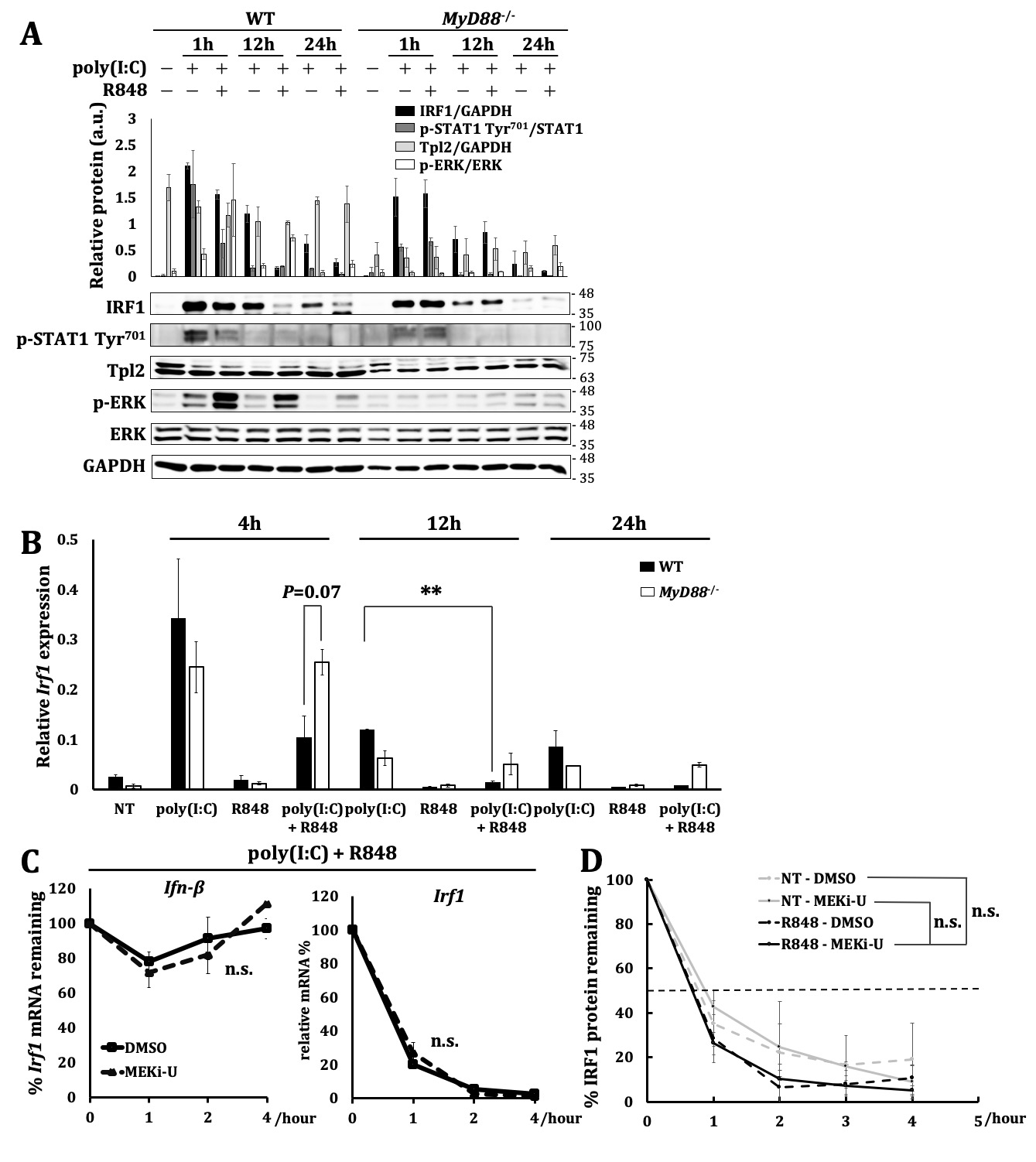


**Fig. S1.** **TLR7 agonist activates MyD88-MEK1/2-mediated suppression in macrophages.** (**A**) Immunoblot analysis and quantitative densitometry for the indicated proteins in the lysate of *Myd88*^-/-^ or WT primary macrophages treated with R848 and/or poly(I:C). Blots are representative of 2 independent experiments. Molecular weight (kDa) markers are indicated on the right side of the blots. Quantified data are means ± SD from all experiments. (**B**) qRT-PCR analysis of *Irf1* mRNA expression in BMDM cells treated with R848 and/or poly(I:C) for indicated time durations. Data are means ± SD pooled from 2 independent experiments. (**C**) qRT-PCR analysis of *Irf1* and *Ifn-β* mRNA stability in R848 and poly(I:C) stimulated J774.1 macrophage cells pre-treated with and without MEKi-U for 4 hours prior to actinomycin D addition for indicated times. Data are means ± SD pooled from 3 independent experiments. (**D**) Quantitative densitometry for IRF1 protein stability in lysate of J774.1 macrophage cells pre-treated with or without MEKi-U for 4 hours prior to cycloheximide addition for indicated time durations. Quantified data are means ± SD from 3 independent experiments. Quantified data are means ± SD from all experiments. **P*<0.05 and ***P*<0.01 by unpaired Welch’s t-test (B and C) or one-way ANOVA (D). n.s, not significant


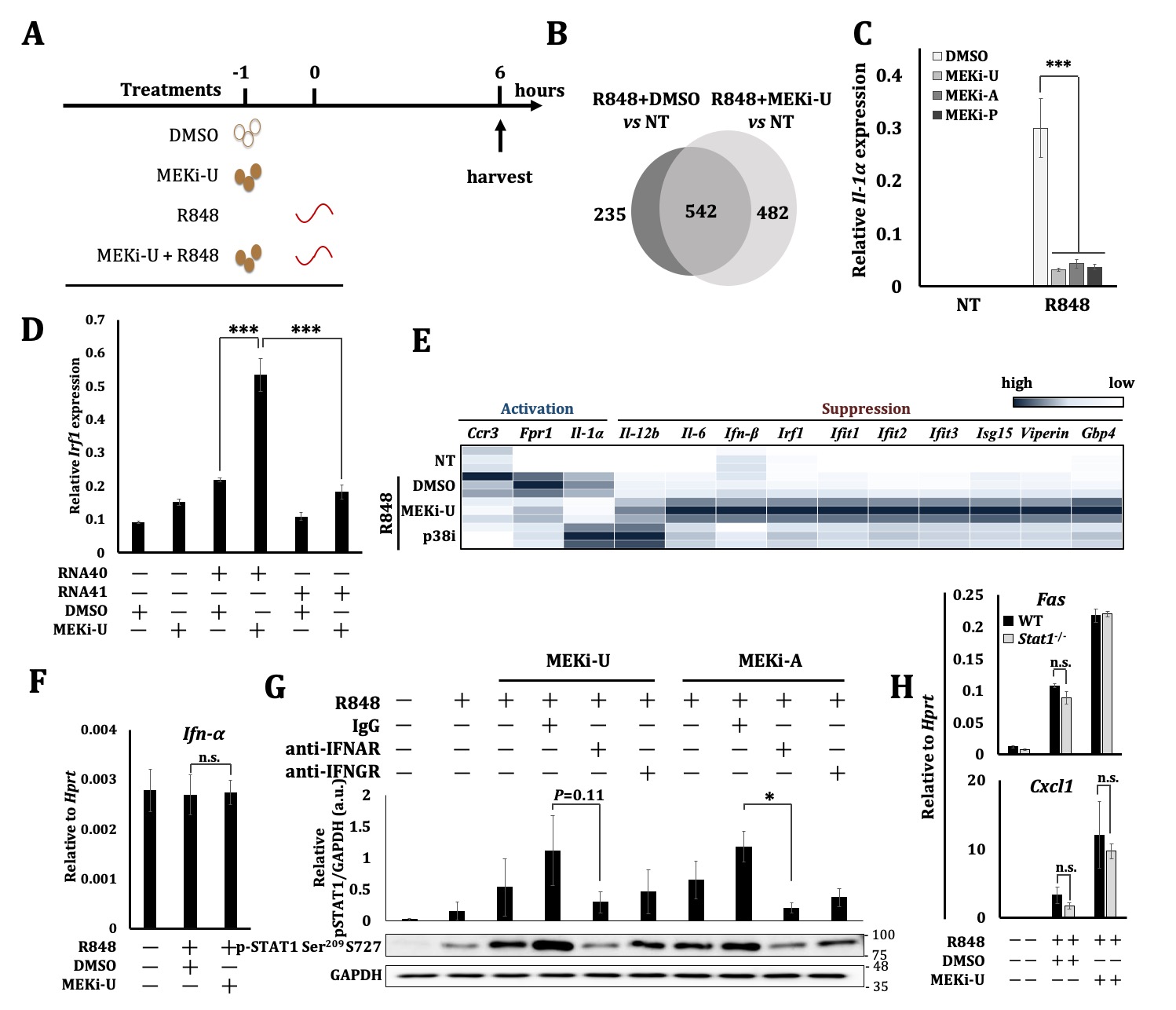


**Fig. S2.** **MEK1/2 inhibitor synergizes with TLR7 agonist in macrophages.** (**A**) treatment scheme for **Fig. 2A**. (**B**) Venn diagram showing numbers of differentially expressed genes from the microarray analysis. (**C**) qRT-PCR analysis of *Il-1α* mRNA expression in BMDM stimulated with R848 for six hours in the presence or absence of MEK1/2 inhibitors as indicated. Data are means ± SD from 4 independent experiments. (**D**) qRT-PCR analysis of *Irf1* mRNA expression in BMDM stimulated with RNA40 or RNA41 for six hours in the presence or absence of MEKi-U. Data are means ± SD from 4 independent experiments. (**E**) heat map showing qRT-PCR analysis of mRNA expression of genes in BMDM stimulated with R848 for six hours in the presence or absence of MEKi-U or p38i. (**F**) qRT-PCR analysis of *Ifn-α.* mRNA expression in BMDM stimulated with R848 for six hours in the presence or absence of MEK1/2 inhibitors as indicated. Data are means ± SD from 4 independent experiments. (**G**) Immunoblot analysis and quantitative densitometry of p-STAT1 S727 in BMDM stimulated with TLR7 agonist R848 for twelve hours in the presence or absence of MEK1/2 inhibitors, IgG isotype control, anti-IFNAR and anti-IFNGR antibodies. Molecular weight (kDa) markers are indicated on the right side of the blots. Blots are representative of 3 experiments. Quantified data are means ± SD from all experiments. (**H**) qRT-PCR analysis of *Fas* and *Cxcl1* mRNA expression in BMDM stimulated with R848 for six hours in the presence or absence of MEK1/2 inhibitors as indicated. Data are means ± SD from 4 independent experiments. **P*<0.05, ***P*<0.01, and ****P*<0.001 by one-way ANOVA (C) or unpaired Welch’s t-test (D, F to H). n.s., not significant.


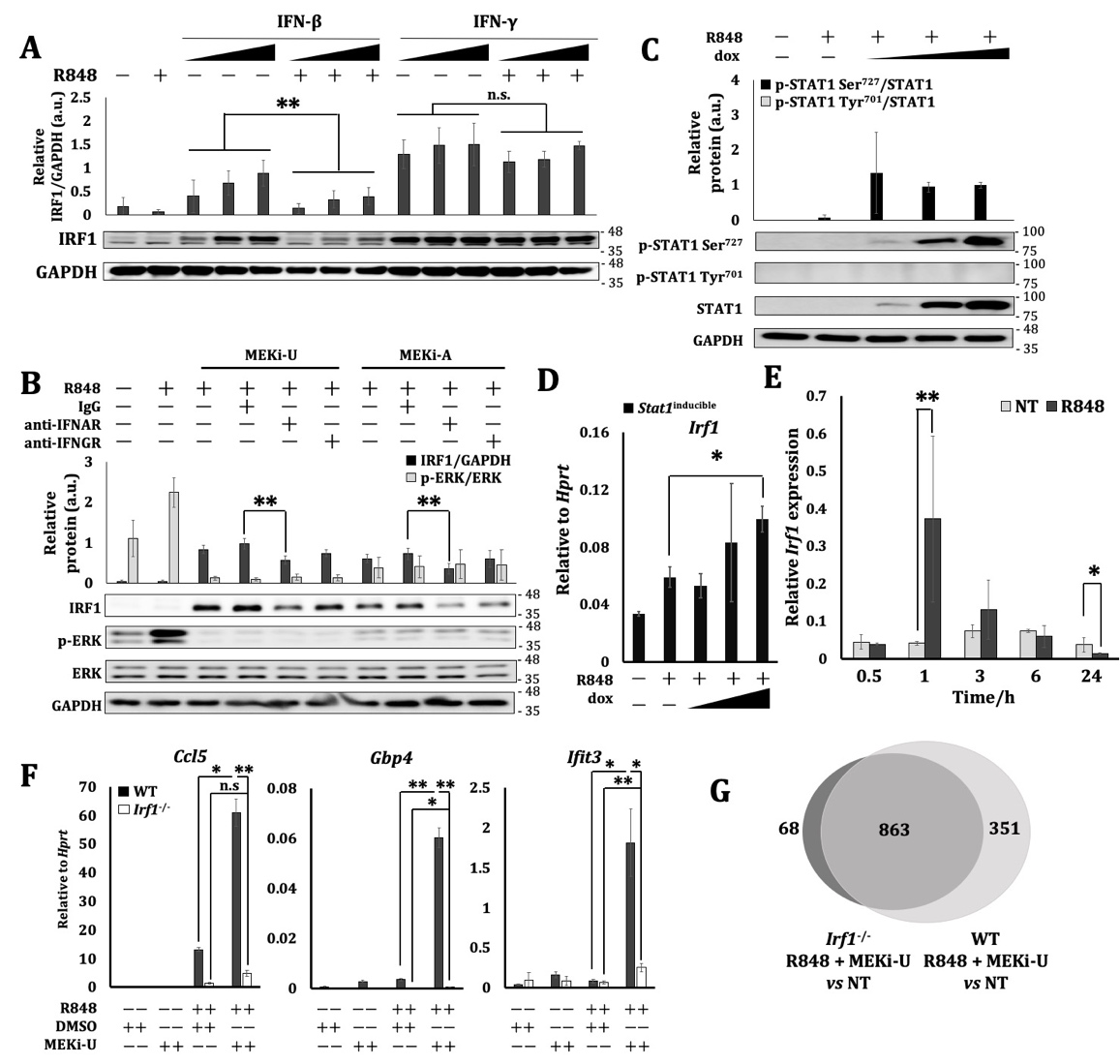


**Fig. S3.** **IRF1 is involved in the unlocked interferon signature response after MEK1/2 inhibition.** (**A**) Immunoblot analysis and quantitative densitometry of IRF1 in J774.1 macrophage cells stimulated with R848 alone, or together with recombinant IFN-β and IFN-γ (2 U/ml, 200 U/ml and 2000 U/ml) for 12 hours as indicated. Blots are representative of 3 independent experiments. Quantified data are means ± SD from all experiments. (**B**) Immunoblot analysis and quantitative densitometry of IRF1, total and p-ERK in BMDM stimulated with R848 for 12 hours in the presence or absence of MEK1/2 inhibitors, IgG isotype control, anti-IFNAR and anti-IFNGR antibodies. Blots are representative of 4 experiments. Quantified data are means ± SD from all experiments. (**C**) Immunoblot analysis and quantitative densitometry of total STAT1, p-STAT1 S727 and p-STAT1 Y701 in *Stat1^inducible^* BMDM pre-treated with increasing concentration of doxycycline (0.03125, 0.125 and 0.5 μg/ml) for 24 hours before addition of R848 for another 8 hours. Blots are representative of 3 experiments. Quantified data are means ± SD from all experiments. (**D**) qRT-PCR analysis of *Irf1* mRNA expression in *Stat1^inducible^* BMDM pre-treated with increasing concentration of doxycycline (0.03125, 0.125 and 0.5 μg/ml) for 24 four hours before addition of R848 for another 8 hours. Data are means ± SD from three independent experiments. (**E**) qRT-PCR analysis of *Irf1* mRNA expression in BMDM stimulated with R848 for indicated time durations. Data are means ± SD from 3 independent experiments. (**F**) qRT-PCR analysis of *Ccl5*, *Gbp4* and *Ifit3* mRNA expression in WT and *Irf1*^-/-^ BMDM treated with R848 for 6 hours in the presence or absence of MEKi-U. Data are means ± SD from 4 independent experiments. (**G**) Venn diagram showing microarray analysis of differentially expressed genes in WT or *Irf1*^-/-^ BMDM. Molecular weight (kDa) markers are indicated on the right side of the blots. **P*<0.05, ***P*<0.01, and ****P*<0.001 by one-way ANOVA (A) or unpaired Welch’s t-test (B, D to F). n.s., not significant.


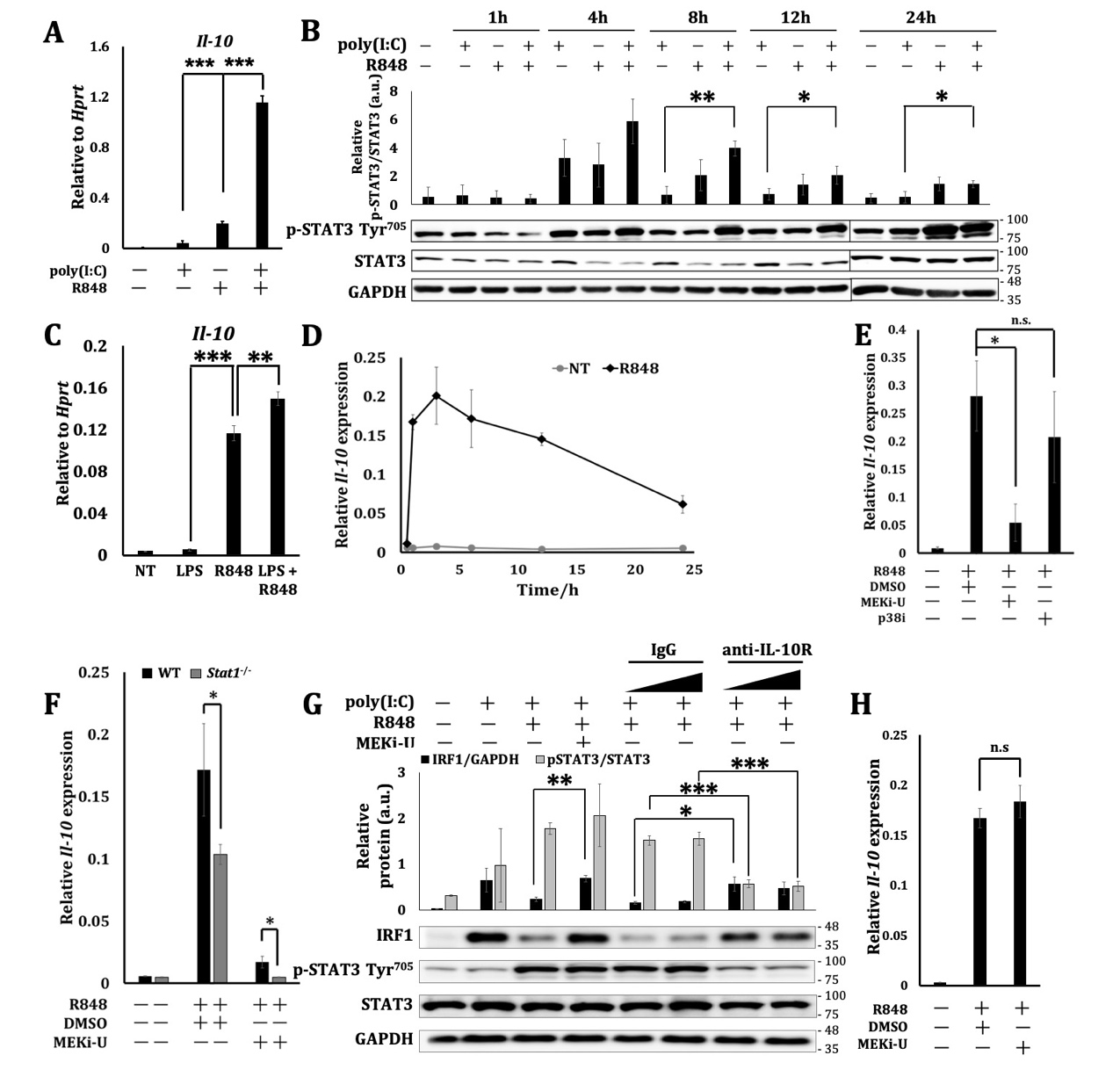


**Fig. S4*.* IL-10 signaling suppresses IRF1-mediated interferon signature response**. (**A**) qRT-PCR analysis of *Il-10* mRNA expression in BMDM stimulated with poly(I:C) and/or R848 for twelve hours. Data are means ± SD from 3 experiments. (**B**) Immunoblot analysis quantitative densitometry of total and p-STAT3 Y705 in J774.1 macrophage cells stimulated with poly(I:C) and/or R848 as indicated. Blots are representative of 3 experiments. Quantified data are means ± SD from all experiments. (**C**) qRT-PCR analysis of *Il-10* mRNA expression in BMDM stimulated with LPS and/or R848 for twelve hours. Data are means ± SD from 3 independent experiments. (**D**) qRT-PCR analysis of *Il-10* mRNA expression in BMDM stimulated with R848 as indicated. Data are means ± SD from 3 independent experiments. (**E**) qRT-PCR analysis of *Il-10* mRNA expression in BMDM stimulated with R848 for 6 hours in the presence or absence of MEKi-U and p38i. Data are means ± SD from 3 independent experiments. (**F**) qRT-PCR analysis of *Il-10* mRNA expression in WT and *Stat1*^-/-^ BMDM stimulated with R848 for 6 hours in the presence or absence of MEKi-U. Data are means ± SD from 3 independent experiments. (**G**) Immunoblot analysis quantitative densitometry of IRF1, total and p-STAT3 Y705 in BMDM stimulated with R848 and poly(I:C) in the presence or absence of MEKi-U, anti-IL-10R antibody and IgG isotype control (1 μg/ml and 10 μg/ml). Blots are representative of 3 independent experiments. Quantified data are means ± SD from all experiments. (**H**) qRT-PCR analysis of *Il-10* mRNA expression in BMDM stimulated with R848 for 1 hour in the presence or absence of MEKi-U. Data are means ± SD from 3 independent experiments. Molecular weight (kDa) markers are indicated on the right side of the blots. **P*<0.05, ***P*<0.01, and ****P*<0.001 by unpaired Welch’s t-test. n.s., not significant.


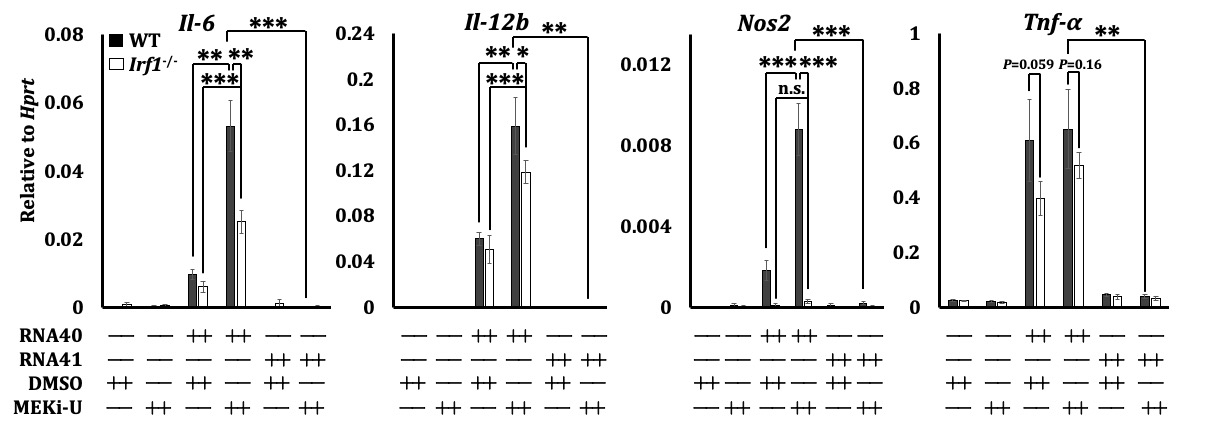


**Fig. S5**. **Combination of MEK1/2 inhibitor and TLR7 agonist modulates macrophage polarization and proliferation**. qRT-PCR analysis of *Il-6*, *Il-12b*, *Nos2* and *Tnf-α* mRNA expression in BMDM stimulated with RNA40 or RNA41 for 6 hours in the presence or absence of MEKi-U. Data are means ± SD from 4 experiments. **P*<0.05, ***P*<0.01, and ****P*<0.001 by unpaired Welch’s t-test. n.s., not significant.
